# Supplementary material for: Netcombin: An algorithm for constructing optimal phylogenetic network from rooted triplets
Source: PLoS One. 2020 Sep 18;15(9):e0227842. doi: 10.1371/journal.pone.0227842 (PMC7500971; doi:10.1371/journal.pone.0227842)
Supplement: S4 File — (ZIP) [file pone.0227842.s004.zip › SequenceData-Triplet/sequenceData-10-20-30-0-Triplet/length 400, numseq 40, rate 10^-7/1 seq.pdf]

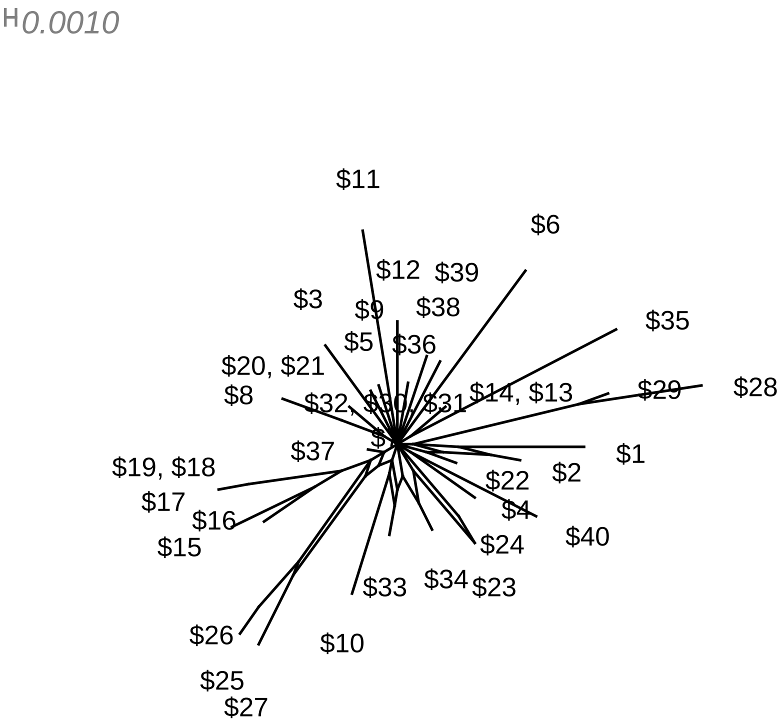
0.0010
$11
$6
$12
$39
$3
$9
$38
$35
$5
$36
$20, $21
$14, $13
$29
$28
$8
$32, $30, $31
$37
$1
$19, $18
$2
$22
$17
$4
$16
$40
$15
$24
$33
$34
$23
$26
$10
$25
$27
